# Quantum Mechanical Versus Polarizable Embedding Schemes: A Study of the Xray Absorption Spectra of Aqueous Ammonia and Ammonium

**DOI:** 10.1021/acs.jctc.4c00105

**Published:** 2024-05-07

**Authors:** Sarai Dery Folkestad, Alexander C. Paul, Regina Paul née Matveeva, Peter Reinholdt, Sonia Coriani, Michael Odelius, Henrik Koch

**Affiliations:** † Department of Chemistry, Norwegian University of Science and Technology, NTNU, 7491 Trondheim, Norway; ‡ Department of Physics, Chemistry and Pharmacy, University of Southern Denmark, SDU, Campusvej 55, 5230 Odense, Denmark; § Department of Chemistry, Technical University of Denmark, DTU, Kemitorvet Bldg 207, 2800 Kongens Lyngby, Denmark; ∥ Department of Physics, Stockholm University, 10691 Stockholm, Sweden

## Abstract

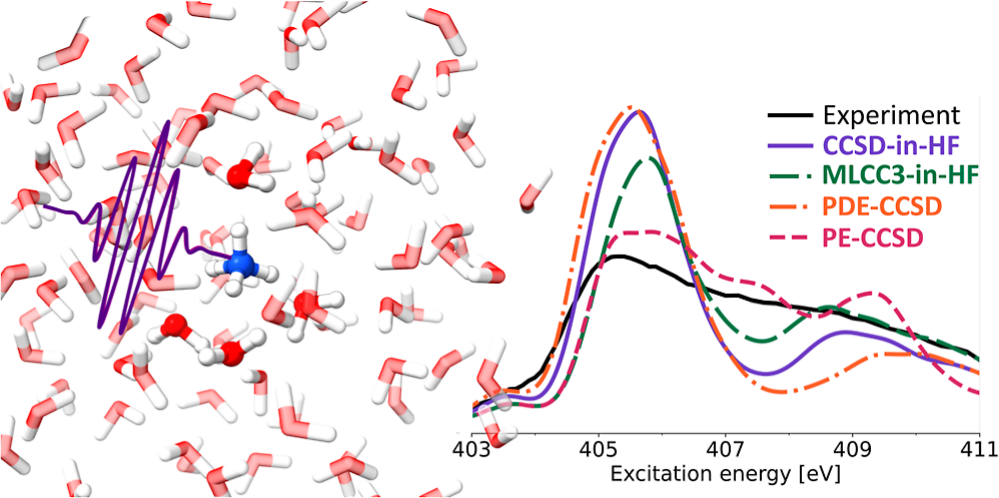

The X-ray absorption spectra of aqueous ammonia and ammonium are computed using a combination of coupled cluster singles and doubles (CCSD) with different quantum mechanical and molecular mechanical embedding schemes. Specifically, we compare frozen Hartree–Fock (HF) density embedding, polarizable embedding (PE), and polarizable density embedding (PDE). Integrating CCSD with frozen HF density embedding is possible within the CC-in-HF framework, which circumvents the conventional system-size limitations of standard coupled cluster methods. We reveal similarities between PDE and frozen HF density descriptions, while PE spectra differ significantly. By including approximate triple excitations, we also investigate the effect of improving the electronic structure theory. The spectra computed using this approach show an improved intensity ratio compared to CCSD-in-HF. Charge transfer analysis of the excitations shows the local character of the pre-edge and main-edge, while the post-edge is formed by excitations delocalized over the first solvation shell and beyond.

## 1 Introduction

Accurate theoretical modeling is crucial for interpreting X-ray absorption (XA) spectra. In gas phase XA experiments on small molecules, a reliable description of the electronic structure of the molecule represents the central theoretical challenge. Coupled cluster theory provides a hierarchy of wave function models to simulate X-ray spectroscopy, i.e., excitations from core orbitals, in small molecular systems.^1^ However, the accuracy depends on the level of coupled cluster theory, and so does the computational cost. When describing core excitations, strong orbital relaxation effects occur, and triple or higher-order excitations in the wave function^1−4^ can be important.

Additional challenges arise when modeling the XA spectra of liquids or solutions: a significant number of representative molecular configurations are needed, and they are inevitably too large for accurate electronic structure models. To overcome the issue, the system is often partitioned and only the target region is treated at a high level of accuracy.^5−7^ However, accounting for solvent effects is crucial, as the solvent can strongly influence the solute’s electronic spectrum. While a high-accuracy treatment is imperative for the immediate surroundings of the solute, the more distant environment can be considered classically or at a lower level of quantum theory. In this paper, we compare three different embedding strategies in the simulation of the XA spectra of ammonia and ammonium in an aqueous solution: the classical polarizable embedding (PE)^8^ model, the semiclassical polarizable density embedding (PDE)^9^ model, and a quantum mechanical embedding in a Hartree–Fock (HF) density.^10,11^

Over the last 50 years, ammonia (NH_3_) and ammonium (NH_4_^+^) have raised the interest of the scientific community for various reasons: one reason is the controversy surrounding their solvation structure.^12^ Another point of interest is the rapid rotation of ammonia in liquid water, which has been under debate for several years.^13^ Ammonia and ammonium are also known in the context of wastewater treatment as the primary forms of inorganic nitrogen.^14^ Moreover, ammonia solution has emerged as a potentially effective CO_2_ absorbent,^15,16^ an important feature in times of increasing environmental pollution.

Ammonia and ammonium have been the subject of numerous experimental and theoretical studies.^12,17−25^ Among these are investigations using XA spectroscopy.^12,23^ Recently, Reinholdt et al.^23^ presented XA spectra of NH_3_ and NH_4_^+^ in water computed using the combination of coupled cluster (CC) methods with PE. The coupled cluster singles and approximate doubles (CC2), and the coupled cluster singles and doubles (CCSD) methods were used. The spectra obtained using CCSD showed satisfactory results and a notable improvement compared to the more commonly used transition-potential density functional theory.^26^ However, as the authors themselves pointed out, it would be beneficial to employ multilevel CC methods and/or extend the number of water molecules in the QM region to validate their conclusions.

In this work, we model the XA spectra of aqueous NH_3_ and NH_4_^+^ using PDE-CCSD and CCSD embedded in a frozen Hartree–Fock density (CCSD-in-HF),^11,27,28^ and compare our results to the PE-CCSD spectra from ref (23). We also examine the effect of including triple excitations in the wave function parametrization using the multilevel coupled cluster singles, doubles, and perturbative triples (MLCC3)^29,30^ embedded in a frozen Hartree–Fock density method (MLCC3-in-HF).

## 2 Theory

The CC wave function is obtained through the exponential ansatz
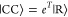
1where the exponential of the cluster operator, *T*, acts on a reference determinant (typically the HF state). The cluster operator generates excitations of the reference, and can be written in terms of excitation operators, τ_μ_, and corresponding amplitudes, *t*_μ_
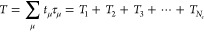
2For instance, single, double, and triple excitations of the reference are generated by *T*_1_, *T*_2_, and *T*_3_, respectively.

The CC energy is obtained by projecting the Schrödinger equation onto the reference determinant
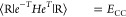
3and the cluster amplitudes are determined by projection onto the space of excited determinants ⟨μ| = ⟨R|τ_μ_^†^
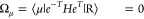
4

In practice, the cluster operator must be truncated at a certain excitation level, which defines the hierarchy of standard CC models. Among the most commonly used models is CCSD, where *T* = *T*_1_ + *T*_2_. In the spin-adapted closed-shell formulation of the theory, we have
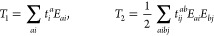
5where *E*_*ai*_ is a singlet excitation operator, and *t*_*i*_^*a*^ and *t*_*ij*_^*ab*^ are the cluster amplitudes. We use indices {*i*, *j*, ...} for occupied orbitals, {*a*, *b*, ...} for virtual orbitals, and {*p*, *q*, ...} for general orbitals.

Excitation energies and transition moments can be calculated with linear response theory or the equation-of-motion^31−33^ (EOM) framework. In EOM CC theory, the ground and excited states are expressed as the eigenvectors of the similarity-transformed Hamiltonian
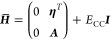
6where  is the Jacobian matrix and . The ground state amplitudes have been determined from eq 4. The *k*’th state is determined by
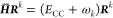
7
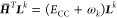
8where ω_*k*_ is the corresponding excitation energy (for *k* > 0). Since ***H*®** is non-Hermitian, the left, ***L***^*k*^, and right, ***R***^*k*^, eigenvectors differ, and so do the state vectors
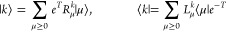
9

We need both left and right state vectors to calculate, e.g., oscillator strengths^34,35^
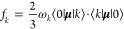
10where |0⟩ is the CC ground state.

Core excitations often have double excitation character, and accurate modeling can rely on the inclusion of triple excitations in *T*. While CCSD scales as  with system size, the exact inclusion of triple excitations, as in CCSDT, will yield -scaling equations, a cost that is prohibitive for most molecular systems. Triple excitations are, therefore, often included perturbatively at an -cost, as in the CC3^36,37^ model.

In this work, we calculate the XA spectra of aqueous ammonia and ammonium. Accurately simulating XA spectra of solutions or liquids requires many representative geometries and a high-level electronic structure description of both solute and neighboring solvent molecules. Hence, a large number of calculations on a sizable molecular system must be carried out, rendering the use of an -scaling model such as CC3 unfeasible.

### 2.1 Multilevel Coupled Cluster Theory

Multilevel coupled cluster (MLCC) theory^7^ can be used to reduce the cost of CC calculations while retaining high accuracy in intensive properties. In MLCC, the highest-order excitations in the cluster operator are restricted to an active space. For instance, in MLCCSDT, *T*_3_ is confined to an active orbital space, whereas *T*_1_ and *T*_2_ include single and double excitations between all occupied and virtual orbitals in the molecular system. The ground and excited states are solved analogously to the standard CC models. However, the projection space is constrained to correspond to the excitations included in *T*. In this paper, we use MLCC3,^29,30^ a multilevel analog of CC3 with triple excitations restricted to an active space.

A similar active space strategy can also define a HF density embedding scheme: by restricting all cluster amplitudes to the active orbital space, we get the CC-in-HF^10,11,38^ models. For example, in CCSD-in-HF, both *T*_1_ and *T*_2_ are restricted. Since high-scaling equations are limited to the active orbital space, we can use CC-in-HF on large molecular systems. Unlike QM/MM approaches, the CC-in-HF models offer a single wave function for the whole molecular system under consideration. Thereby, we include Pauli-repulsion between the target (active region) and environment (rest of the system) regions. In the CC-in-HF scheme, an effective Fock matrix is used to include the interactions with the environment in the CC calculation
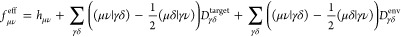
11

Here, *h*_μν_ are the one-electron integrals in the atomic orbital (AO) basis, (μν|γδ) are the electron repulsion integrals in Mulliken ordering, and *D*_γδ_^target^ and *D*_γδ_^env^ are the active and environment densities, respectively. The total density
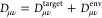
12is optimized at the HF level of theory, and the partitioning of the orbital space occurs between the HF and CC calculations. Note that all AOs—both of the target region and the environment—can contribute to the active molecular orbitals (MOs), i.e., the AO indices μ and ν are not restricted to the target region in eq 11. Conversely, AOs centered on the active region can contribute to the environment density. A reduced dimension of the effective Fock matrix is only obtained through transformation to the active MO basis. The inactive occupied orbitals contribute to the reduced-space MO-Fock matrix through ***D***^env^ in the last term of eq 11.

Finally, we can combine the MLCC and the CC-in-HF approaches to obtain the MLCC-in-HF model. This can be done by introducing two concentric active spaces, where all *t*-amplitudes are restricted to the outer active space, and the high-order *t*-amplitudes are limited to the inner space.^11^

Successful application of the CC-in-HF and MLCC approaches depends on the partitioning of the orbital space. We need to choose a suitable type of HF orbitals and determine the size of the active space. For the XA spectra of ammonia and ammonium, we select the outer active orbital layer using localized orbitals. This ensures that the solute and a fixed number of solvent molecules are included in the correlated part of the calculation. Any kind of localized HF orbitals can be used. In this work, we use Cholesky occupied orbitals^10^ and orthonormalized projected atomic orbitals^39,40^ (PAOs) for the virtual space. The Cholesky orbitals result from a partial Cholesky factorization of the HF density in the AO basis
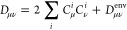
13

The elements of the Cholesky vector, ***C***^*i*^, are the orbital coefficients of the active occupied orbital *i*. The partial factorization is obtained by restricting the pivots of the decomposition to the AOs of the target region. Furthermore, to ensure a tight active space, we exclude pivoting elements corresponding to diffuse orbitals with exponents smaller than or equal to 0.6.

For the virtual space, active orbitals are constructed by projecting the occupied orbitals out of the AOs centered on the target region
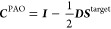
14

The overlap matrix ***S***^target^ is rectangular and contains the columns of the AO overlap matrix that correspond to AOs centered on the active atoms. Before the CC calculation, we remove linear dependencies and orthonormalize the virtual orbitals.

For the inner active space in the MLCC3 calculations, we use correlated natural transition orbitals^41^ (CNTOs). The CNTOs are constructed from CCSD (or CCSD-in-HF) excitation vectors, ***R***^*k*^, by diagonalizing the unit-trace matrices
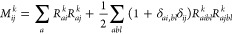
15and
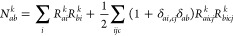
16and transforming the occupied and virtual blocks of the orbital coefficient matrix with the resulting eigenvector matrices. Using CNTOs, a compact representation of the excitation captured by ***R***^*k*^ can be achieved. To obtain an orbital space that targets a collection of excited states, the matrices ***M*** = ∑_*k*_***M***^*k*^ and ***N*** = ∑_*k*_***N***^*k*^ are diagonalized. The resulting eigenvector matrices are used as transformation matrices for the occupied and virtual orbital coefficients, respectively. Considering several excited states simultaneously will increase the required size of the CNTO active space. However, an expanded active space often results in a higher accuracy in the calculated excitation energies. The quality of the CNTOs depends on how well the CCSD model describes the transitions. Therefore, transitions with significant double excitation character are described less accurately in the CNTO basis. However, as long as the active space is sufficiently large, MLCC3 is able to recover doubly excited states as well, although at lower accuracy.

### 2.2 Polarizable Density Embedding

The PDE^9^ scheme shares similarities with the HF density embedding outlined above, and is an extension of the PE approach by Olsen et al.^8^ In PDE, contributions from the environment are added to the Fock matrix of the target system according to
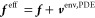
17

The embedding operator contains three terms, accounting for the electrostatic (es), nonelectrostatic repulsion (rep), and induction (ind) interactions
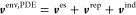
18

The electrostatic term includes the effects of the nuclear-electron attraction and the electron–electron repulsion between electrons in the target system and the nuclei and electrons of the environment; the latter of which is included through frozen fragment densities, ***D***^*f*^, of the environment molecules
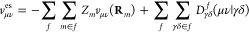
19

Here, *Z*_*m*_ is the charge of nucleus *m* in fragment *f*, *v*_μν_ is a nuclear attraction integral, *D*_γδ_^*f*^ is the density matrix of environment fragment *f*, and (μν|γδ) are (intermolecular) electron repulsion integrals.

Similarly to the CC-in-HF approach, the PDE model retains the “exact” electron–electron repulsion from the electronic densities of the environment rather than approximating this interaction with a distributed multipole model, as is done in the simpler PE models. However, unlike in CC-in-HF, the environment density in PDE is made up of frozen fragment densities that are calculated in isolation;^9^ in CC-in-HF the total density (***D*** = ***D***^target^ + ***D***^env^) is optimized at the HF level of theory. As a result, the total HF density  is idempotent in CC-in-HF, which is not the case for PDE.

Compared to PE, the PDE potential also includes Pauli-repulsion through a Huzinaga–Cantu-style^42^ repulsion operator
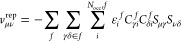
20which penalizes the wave function overlap between the target region and the environment fragment wave functions. The repulsion operator depends on the environment fragment orbital energies ε_*i*_^*f*^, MO coefficients *C*_γ*i*_^*f*^, and the intermolecular overlap integrals *S*_μγ_. This can be compared to the exchange contribution to the target system Fock matrix in CC-in-HF (see eq 11).

Finally, the PDE models include mutual polarization effects between the target region and the environment which is described by an induced dipole model, as in PE.^8^ The induction operator appears as
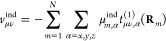
21where μ_*m*,α_^ind^ is a component of an induced dipole moment and *t*_μν,α_^(1)^ are matrix elements of the electric field operator from the QM region. The induced dipoles are obtained as solutions to the linear problem
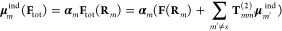
22where **F** is the electric field, and **T**^(2)^ is the dipole–dipole interaction tensor. Thus, the polarizability α_*m*_ creates an induced dipole in response to the electric fields from the QM density, the static fields from all other environment fragments, and the fields from every other induced dipole moment in the environment. As a result, the induced dipoles depend on the QM density, which in turn depends on the values of the induced dipoles, ensuring mutual polarization between the environment and the QM density.

### 2.3 Charge Transfer Analysis of Excitations

When a molecular system absorbs a photon, the electrons rearrange themselves, causing a change in density
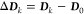
23where ***D***_*k*_ is the density of an excited state and ***D***_0_ the ground state density. The trace of the one-electron density equals the number of electrons. Therefore, the trace of the density difference, Δ***D***_*k*_, must be zero. Using, for example, Löwdin population analysis, we can transform the density difference from the delocalized MO basis to the local AO basis
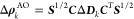
24where ***S*** is the AO overlap matrix and ***C*** contains the MO-coefficients. Since ***C***^*T*^***SC*** = ***I***, the trace of the density difference remains zero in the AO basis. We can now partition the trace of Δ**ρ**_*k*_^AO^ into contributions from the AOs on different fragments of the molecular system. For instance, for the calculation of XA spectra of a solvent or liquid, we can partition the trace with respect to (A) AOs on the solute, (B) AOs on the water molecules closest to the solute, and (C) the rest of the AOs
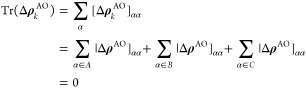
25

The trace of a single subsystem is then interpreted as the number of electrons detached from or attached to this subsystem, depending on the sign. Such analysis has been reported before for TDDFT using Mulliken population analysis in ref (43) and more generally in ref (44).

## 3 Results and Discussion

Using PDE-CCSD and CC-in-HF, we describe the solute and four closest water molecules with CC theory, whereas the remaining solvent molecules are described at a lower level of theory. These methods serve to reduce the high cost associated with applying CC methods to large systems without compromising the accuracy in intensive molecular properties. The CC-in-HF calculations are performed with a development version of the  program,^27^ while the PDE-CCSD calculations are carried out with the Dalton program^45^ (version 2022), and we use the 195 spherical cluster geometries studied in ref (23). For the PDE,^9^ electron densities at the CAMB3LYP^46^/aug-cc-pVTZ level are computed for each molecule, except the solute and four closest water molecules. The embedding potential is constructed from these fragment densities and atomic polarizabilities, obtained with the LoProp^47^ method. The spectra are calculated from a CCSD wave function optimized in the presence of the embedding potential. For CC-in-HF, the orbitals localized on the solute and the four closest water molecules are included in the CC region. The remaining orbitals are described using a frozen HF density.^10,11,27^ We use two versions of CC-in-HF: CCSD-in-HF and MLCC3-in-HF, which are described in more detail in ref (28). In all calculations the core–valence separation approximation^48−50^ is used to target core excitations.

**Figure 1:**
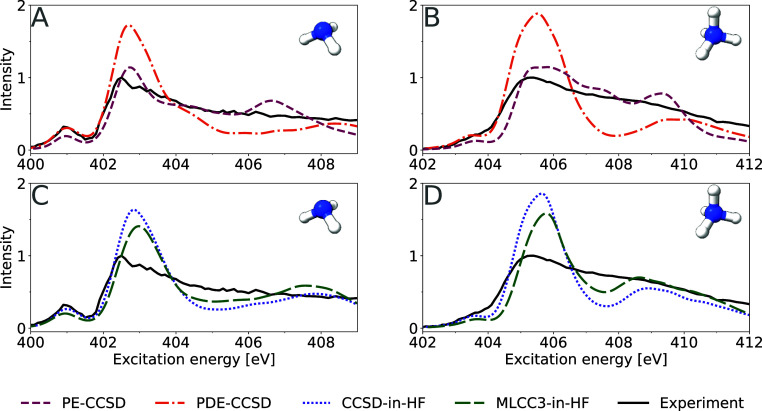
XA spectra of ammonia (panels A, C) and ammonium (panels B, D) in water clusters for different embedding methods. Solute and four closest water molecules are described with 6-311++G** basis, while remaining solvent molecules are treated at the PE, PDE, and HF/6-31G** levels of theory. The PE-CCSD, PDE-CCSD, CCSD-in-HF, and MLCC3-in-HF results have been shifted by −1.9, −2.8, −2.5, −1.5 eV, respectively. The PE-CCSD data was replotted based on calculations from ref (23), and the experimental data was digitized from ref (12) using Web Plot Digitizer.^51^

To compare with the study of Reinholdt et al.,^23^ we performed calculations using the same basis set (6-311++G**) in the CC region. The remaining water molecules were described using either PDE or HF embedding with the 6-31G** basis set. The resulting spectra for both ammonia and ammonium are presented in Figure 1. We use a constant broadening with Voigt profiles of 0.2 eV Lorentzian full width at half-maximum (fwhm) and Gaussian standard deviation (yielding a total fwhm of ∼0.59 eV) to obtain smooth theoretical spectra. The average spectra are normalized such that the area underneath the curve equals that of the experiment in the ranges 400–408 eV and 402.5–411 eV for NH_3_ and NH_4_^+^, respectively. The theoretical spectra for ammonia were shifted to align approximately with the pre-edge of the experiment (see Figure 1). For ammonium the same shifts were applied.

Comparing the theoretical spectra of ammonia and ammonium, shown in Figure 1, we observe that the spectral shapes of the PE-CCSD spectra agree better with experiment compared to both PDE-CCSD and CCSD-in-HF. Since both the PDE and HF embedding schemes offer a higher level of theoretical description of the solvent molecules, the favorable agreement of PE-CCSD with experiment is likely due to cancellation of errors. For instance, a density-based embedding scheme includes the effects of Pauli-repulsion, which is not the case for PE. We note that PDE-CCSD and CCSD-in-HF exhibit very similar spectral features. Both methods overestimate the ratio between the main-edge (at 402.8 eV) and the post-edge (403 to 409 eV) of the ammonia spectrum (see panels A and C of Figure 1), compared to experiment and PE-CCSD.

The main-edges in PE-CCSD and PDE-CCSD exhibit a shoulder at approximately 404.5 eV, a feature not observed for CC-in-HF. It has been shown for a water dimer that PDE is overestimating the repulsion energy compared to HF.^9^ Therefore, in CC-in-HF, the transitions in this energy region are probably shifted to lower energies so that the shoulder merges with the main-edge. With the selected normalization scheme, the intensity of the post-edge region is underestimated by PDE and HF-embedding, except for a very broad feature at ∼408 eV. This broad band is shifted to lower energies for CCSD-in-HF compared to PDE-CCSD. In contrast to PE-CCSD, we do not observe a spectral feature at the ionization limit at approximately 407 eV. Similar to the study by Reinholdt et al.,^23^ we do not treat the continuum differently than the other regions of the spectrum. Therefore, we conclude that this spectral feature is more likely an artifact of the PE description, rather than the result of the discretized description of the continuum.

For aqueous ammonium (see panels B and D of Figure 1), the important spectral characteristics—the shoulder between 403 and 404 eV, the main-edge at 405.7 eV, and the post-edge between 407 and 412 eV—are reproduced with PDE-CCSD and CCSD-in-HF. However, compared to PE-CCSD, the transition from main- to post-edge is characterized by a significant drop in intensity. This intensity decrease is more pronounced for PDE-CCSD, because the broad post-edge feature is shifted to higher energies compared to CCSD-in-HF. While the PE-CCSD spectrum agrees best with the experiment, it contains a rather sharp feature in the range 408 to 410 eV which is much broader in the other embedding schemes. This additional peak resembles the one in the ammonia PE-CCSD spectrum, and further supports that it might be an artifact of the PE.

**Figure 2:**
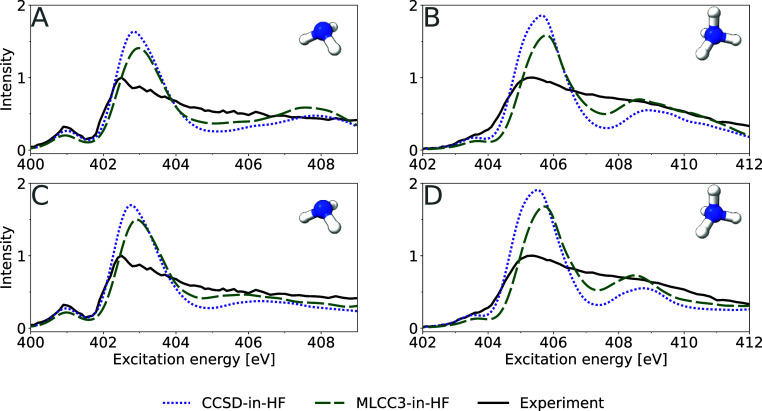
Comparison of XA spectra computed using Pople (panels A, B) and Dunning (panels C, D) basis sets. XA spectra of ammonia (panels A, C) and ammonium (panels C, D) in water clusters at the CCSD-in-HF and MLCC3-in-HF levels of theory. In the upper row 6-311++G** was used in the CCSD space and 6-31G** for the remaining solvent, while in the lower row aug-cc-pVTZ was used for the solute, aug-cc-pVDZ for the four closest water molecules and cc-pVDZ for the remaining solvent. Constant energy shifts of −2.5 and −2.0 eV were applied for CCSD-in-HF and −1.5, −1.0 eV for MLCC3-in-HF for the Pople and Dunning bases, respectively.

In Figure 2, we show the effect of including approximate triple excitations and changing the basis sets for the XA spectra of ammonia and ammonium in aqueous solution. Comparing MLCC3-in-HF to CCSD-in-HF, we see that inclusion of approximate triple excitations yields an improved intensity ratio between the main- and post-edge in the ammonia spectrum, resulting in a smoother transition between the two regions. The absolute peak positions in MLCC3-in-HF are more accurate than in CCSD-in-HF. Similarly, the relative intensities of the main- and post-edge are improved going from CCSD-in-HF to MLCC3-in-HF for ammonium: in the MLCC3-in-HF spectrum, the main-edge intensity is reduced and the post-edge intensity is increased. Additionally, we observe a red-shift of the post-edge feature from CCSD-in-HF to MLCC3-in-HF.

To examine the basis set effect on the spectra, we test various basis set combinations for the solute, the closest four waters and the remaining solvent molecules on an arbitrarily chosen snapshot. The results are shown in Figures S1 and S2 in the Supporting Information. A combination of aug-cc-pVTZ on the solute, aug-cc-pVDZ on the four closest water molecules, and cc-pVDZ on the remaining solvent molecules was chosen as a compromise between accuracy of the basis and computational efficiency. Using these basis sets, the XA spectra of both ammonia and ammonium were recomputed and compared with previous spectra (see Figure 2). For both ammonia and ammonium, the change of basis set leaves the pre- and main-edges of the spectra unchanged. However, for ammonia, the post-edge feature is broadened and red-shifted when the Dunning basis sets are used. This change in spectral shape shows that the post-edge feature of ammonia observed using the Pople basis is not a real resonance, but rather an artifact of a limited basis set. For ammonium, on the other hand, the post-edge feature around 408.5 eV does not shift, and compression of the spectrum with increased basis set size is only observed above 409 eV.

**Figure 3:**
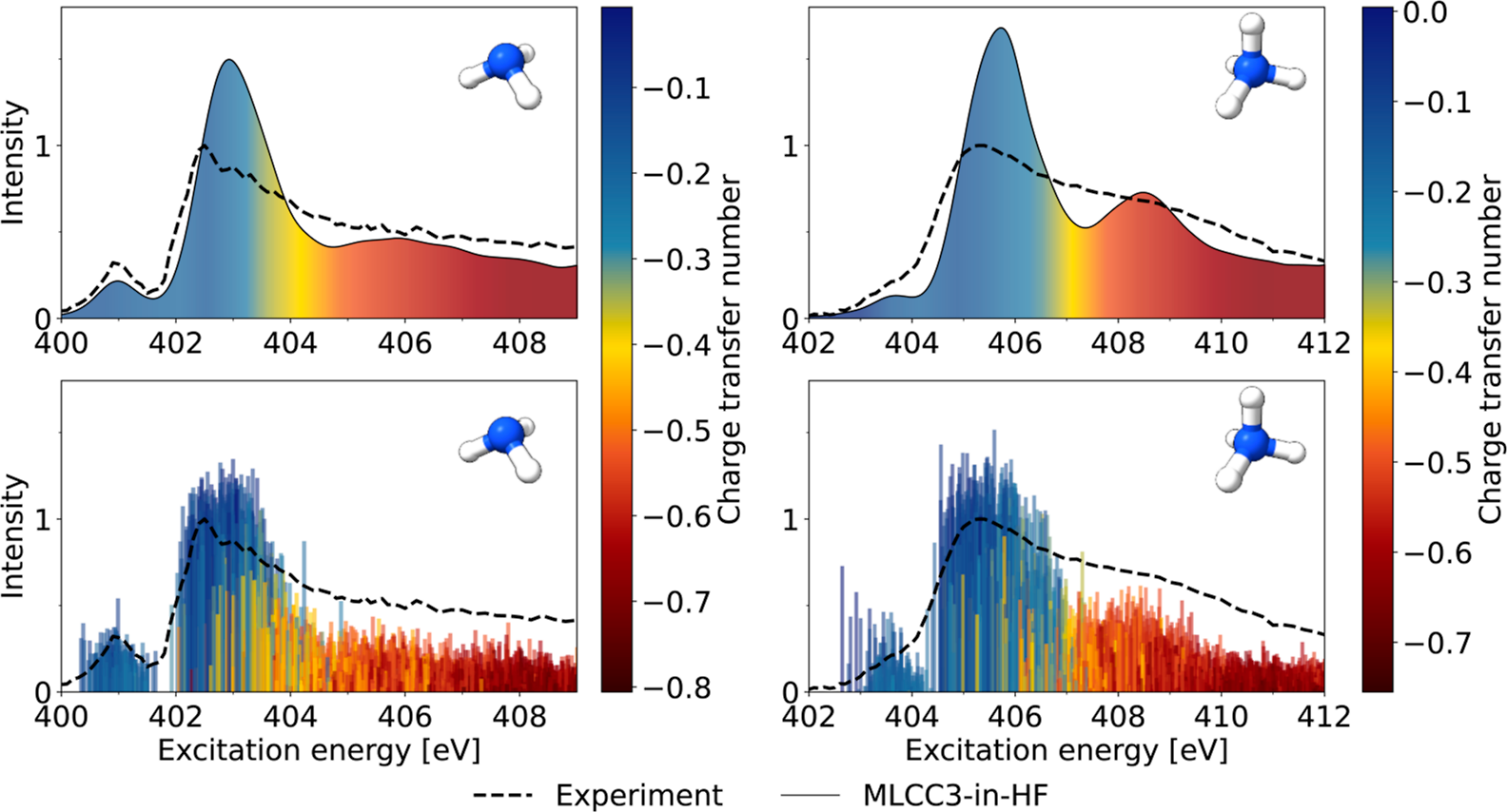
XA spectrum plotted together with the CT number calculated for the solute molecules (ammonia, left column) and (ammonium right panel). In the lower panel, each excitation has been assigned a color based on its CT character. In the upper panel, we show the broadened spectrum where the color gradient indicates the CT character averaged for all transitions within a bin of the size equal to the fwhm.

In Figure 3, we present a charge transfer (CT) analysis for the calculated MLCC3-in-HF excitations of the aqueous ammonia and ammonium solutions. This analysis is based on the calculation of the so-called CT number for each excited state of the solute molecule.^43,44,52,53^ The CT number shows the significance of the interaction with the solvent. A negative value indicates the removal of electrons from the solute while a positive value implies their addition. As described above, and evident from the similarity in the respective values for NH_3_ and NH_4_^+^, the CT number acts as an indicator of environmental influence rather than actual electron transfer. From Figure 3 we see that the excitations in the pre- and main-edge in the XA spectra of both molecular systems are localized on the respective solute molecules. In the post-edge regions, we observe an increasing CT indicating larger influence from the solvent molecules. This reflects the more diffuse nature of excitations in these regions.

In Figure S3 in the Supporting Information we show the CT from the solute and four surrounding water molecules to the remaining solvent. We see that the excitations responsible for generating the pre-edge, main-edge, and the first half of the post-edge are well described by the four water molecules in the CC region. An increasing degree of CT in the second half of the post-edge is observed. However, it is important to note that the CT analysis is only performed on the orbitals within the CC region. Consequently, effects beyond the four nearest water molecules can only be included through the diffuse basis functions and at the HF level.

To ensure the comparability with the results by Reinholdt et al.,^23^ we have included only four water molecules in the CC region. However, it is natural to increase the number of solvent molecules in the CC region to examine the potential improvement in the spectra. This is particularly interesting in the case of ammonium, where the number of water molecules in the first solvation shell is known to be 5.2.^54^ We have calculated the spectra of aqueous ammonium and ammonia with six water molecules in the CC region for a subset of 30 snapshots. The results are presented in Figures S4 and S5 in the Supporting Information. Although indicating that increasing the CC region could improve the imbalance between the intensities of the main and post edges, no significant changes in the spectra are observed. These results are in agreement with the CT analysis (see Figure S3), indicating that the CC region is sufficiently large. However, a further improvement of the electronic structure, achievable through a higher level of CC theory, or improved quality of the structures can further reduce the discrepancy between theory and experiment. The quality of the structures can, for example, be improved by accounting for nuclear quantum effects in the molecular dynamics simulations, which play an important role in capturing the delicate nature of hydrogen bonds.^55^ In a recent study on the XA of liquid water,^28^ we have observed both a redistribution of intensity from the main- to the post-edge and a broadening effect from the inclusions of NQEs. These effects might be more pronounced in both ammonia and ammonium solutions because the hydrogen bonds are weaker compared to water.^12^

## 4 Conclusions

We have compared the performance of the different embedding schemes—PE-CCSD, PDE-CCSD, CCSD-in-HF and MLCC3-in-HF—in simulating the XA spectra. Our comparison reveals similarities between PDE-CCSD and CCSD-in-HF, while PE-CCSD results differ noticeably. Given that PDE-CCSD represents a theoretical refinement of PE-CCSD^9,56^ and CC-in-HF methods are fully quantum mechanical, we conclude that the close agreement of PE-CCSD with experiment is due to error cancellation. Approaches that treat the environment through a density-type embedding will avoid issues relating to the lack of Pauli-repulsion, as is a concern with PE.

A CT analysis demonstrates the local character of the excitations responsible for the pre- and main-edge, in contrast to the more diffuse nature of post-edge excitations in both solutions. Exploring the impact of an increased number of solvent molecules in the CC region did not provide any significant improvement. Therefore, we conclude that our methods offer a robust description of the electronic structure of the solute, with potential for improvement by using state-of-the-art path integral molecular dynamics trajectories or through higher-level electronic structure models.

## Data Availability

Geometries are available from 10.5281/zenodo.10390676. The data sets generated and analyzed during the current study are available from the corresponding author on reasonable request.

## Supporting Information Available

The Supporting Information contains an examination of the basis set requirements on one arbitrarily selected snapshot, CT analysis on the solute and the four closest solvent molecules, XA spectra obtained using various broadening schemes (Gaussian and Lorentzian), comparison of XA spectra obtained when four vs six water molecules are included in the CC region (PDF)
