# Supplementary material for: Quantum Mechanical Versus Polarizable Embedding Schemes: A Study of the Xray Absorption Spectra of Aqueous Ammonia and Ammonium
Source: J Chem Theory Comput. 2024 May 7;20(10):4161–9. doi: 10.1021/acs.jctc.4c00105 (PMC11137810; doi:10.1021/acs.jctc.4c00105)
Supplement: Supplementary file 1 — ct4c00105_si_001.pdf [file ct4c00105_si_001.pdf]

Supporting Information: Quantum Mechanical versus Molecular Mechanical Embedding Schemes: A Study of the X-ray Absorption Spectra for Aqueous Ammonia and Ammonium

Sarai Dery Folkestad,[†,‖] Alexander C. Paul,[†,‖] Regina Paul (née Matveeva),[†,‖] Peter Reinholdt,[‡] Sonia Coriani,[¶] Michael Odelius,[§] and Henrik Koch[*,†]

†*Department of Chemistry, Norwegian University of Science and Technology, NTNU, 7491 Trondheim, Norway*

‡*Department of Physics, Chemistry and Pharmacy, University of Southern Denmark, SDU, Odense, Denmark*

¶*Department of Chemistry, Technical University of Denmark, DTU, 2800 Kongens Lyngby, Denmark*

§*Department of Physics, Stockholm University, 10691 Stockholm, Sweden*

‖*These authors contributed equally to this work.*

E-mail: henrik.koch@ntnu.no

Basis Set Study

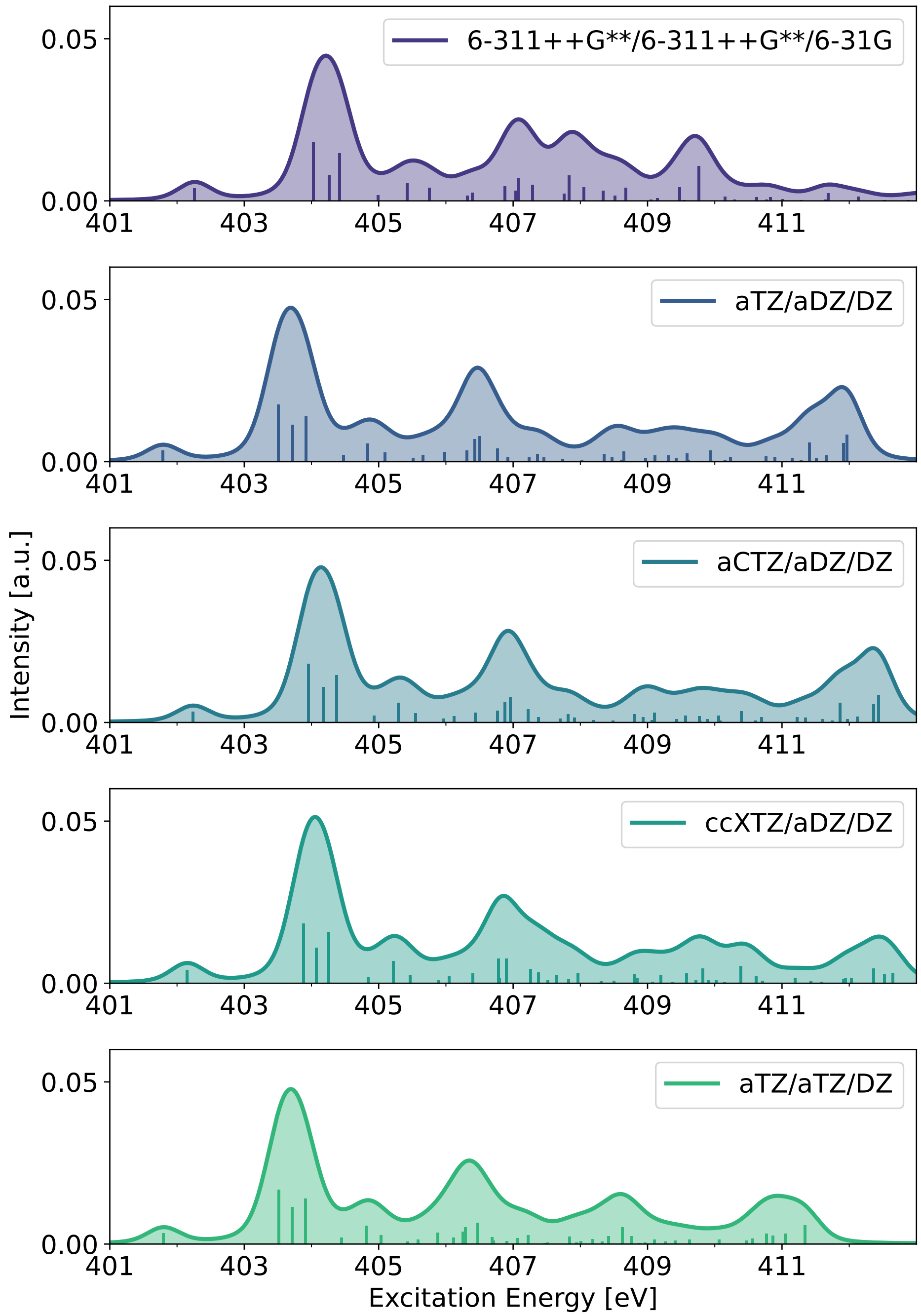

Figure S1: Comparison of different basis sets for the XA spectrum of **ammonia** in water at the MLCC3-12/72 level of theory using a single snapshot of the dynamics simulation (step 2200). Individual excitations have been broadened using Voigt profiles with 0.2 eV Lorentzian fwhm and 0.2 eV Gaussian standard deviation (total fwhm ∼0.59 eV).

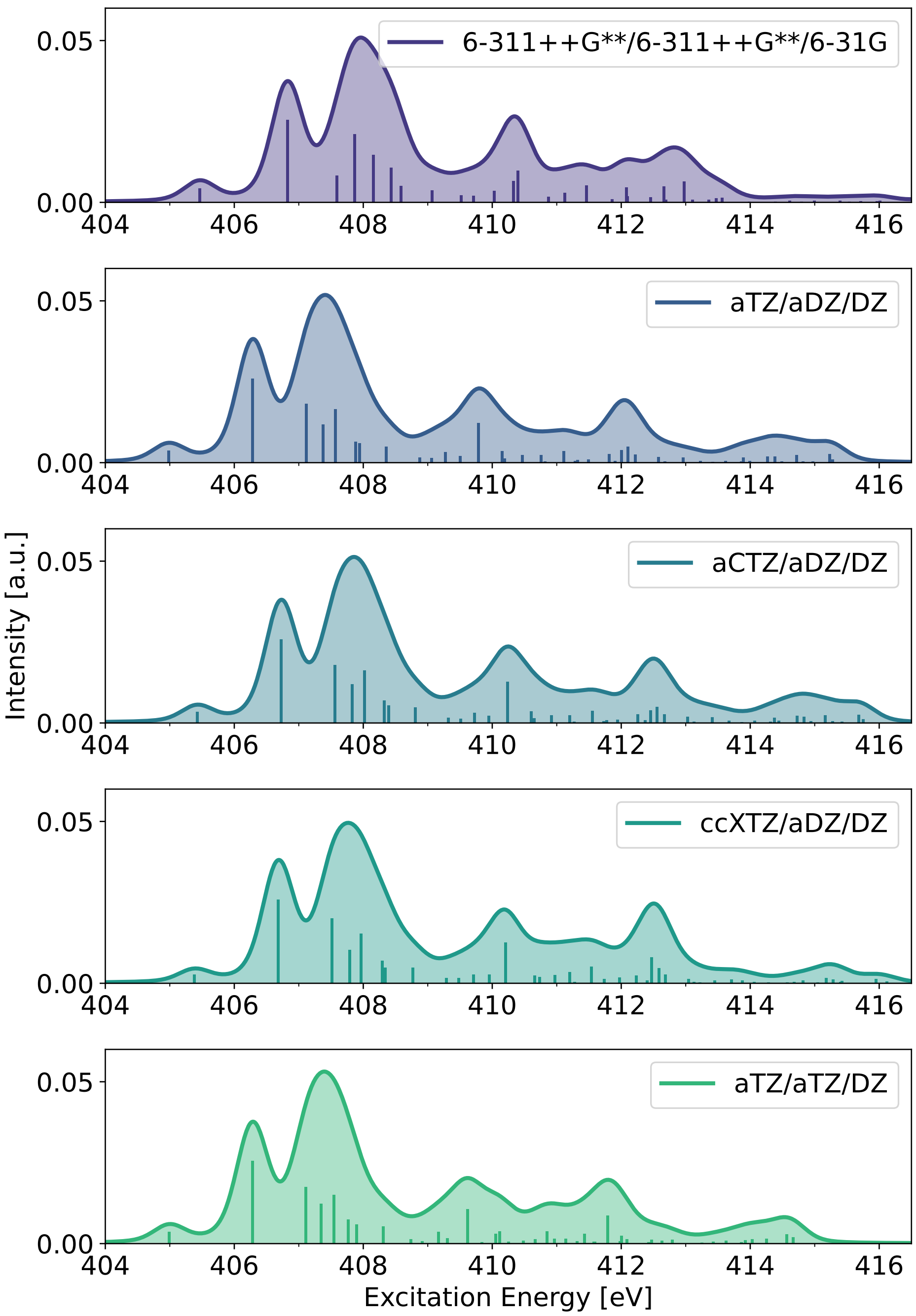

Figure S2: Comparison of different basis sets for the XA spectrum of **ammonium** in water at the MLCC3-12/72 level of theory using a single snapshot of the dynamics simulation (step 2200). Individual excitations have been broadened using Voigt profiles with 0.2 eV Lorentzian fwhm and 0.2 eV Gaussian standard deviation (total fwhm ∼0.59 eV).

Charge Transfer Analysis

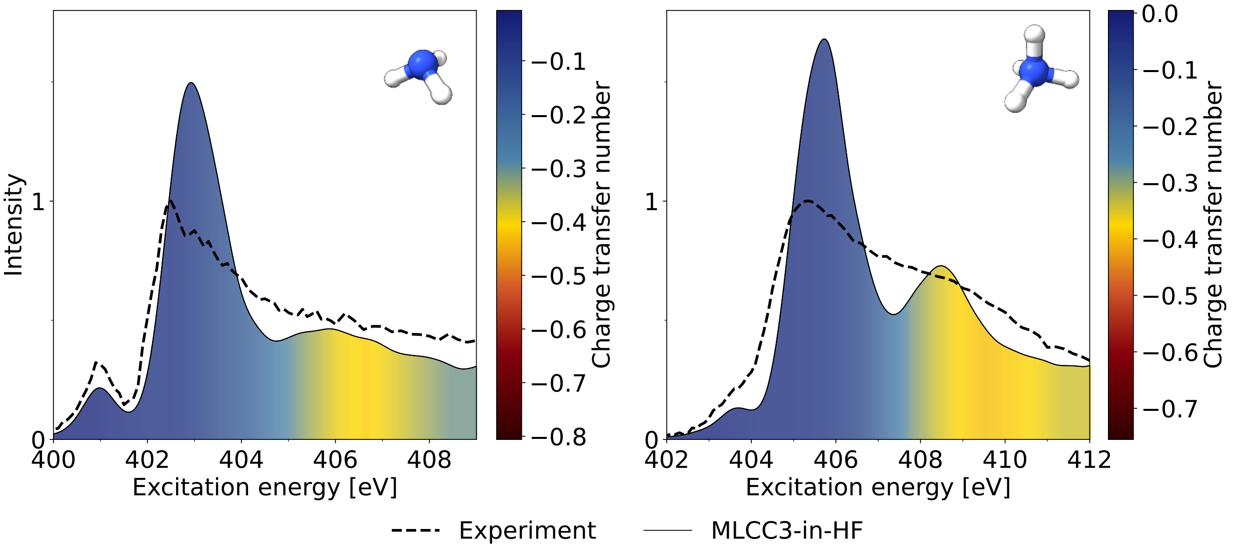

Figure S3: Spectrum plotted together with the charge transfer number for charge transfer from the central molecule and the 4 closest water molecules (ammonia (left) and ammonium (right)). The color map is identical to the one in Figure 3 of the main paper.

Increasing the number of solvent molecules in CC region

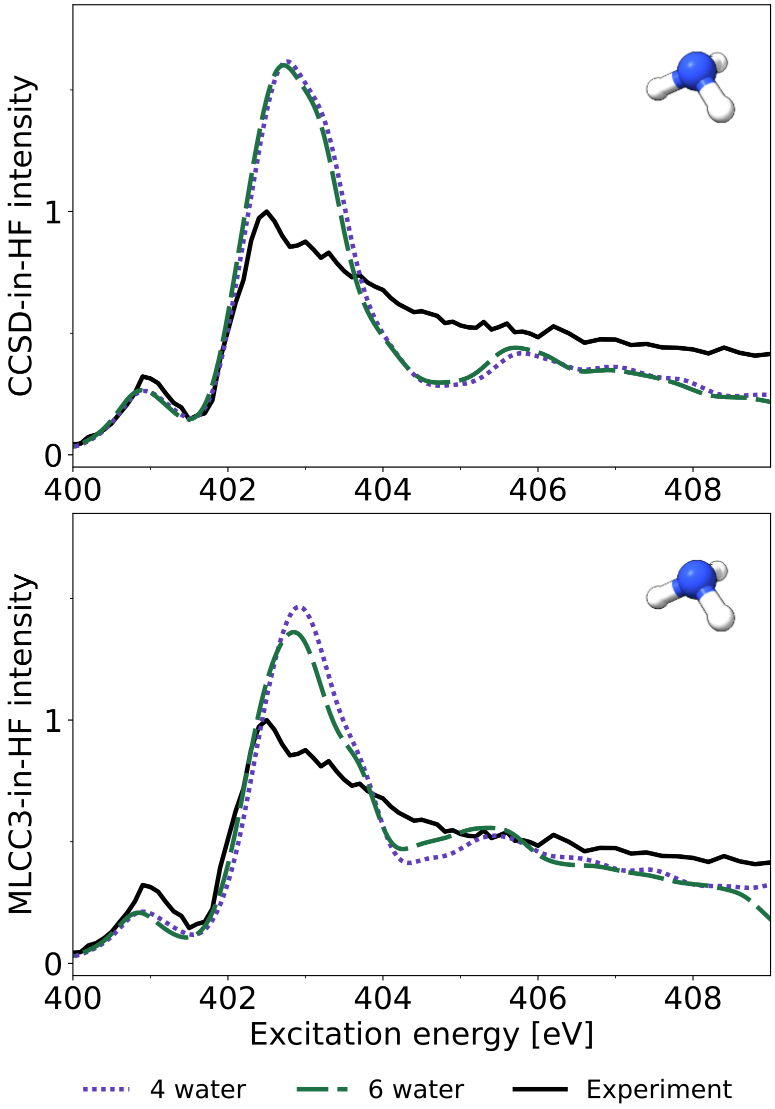

Figure S4: The effect of including 6 closest water molecules in CC-in-HF calculations for NH_3. Average over 30 snapshots. Voigt profiles with Lorentzian fwhm of 0.2 eV and 0.2 Gaussian standard deviation. XA spectra of **ammonia** in water clusters at the CCSD-in-HF and MLCC3-in-HF level of theory. The spectra have been shifted by $-2\,\text{eV}$ and $-1\,\text{eV}$, respectively.

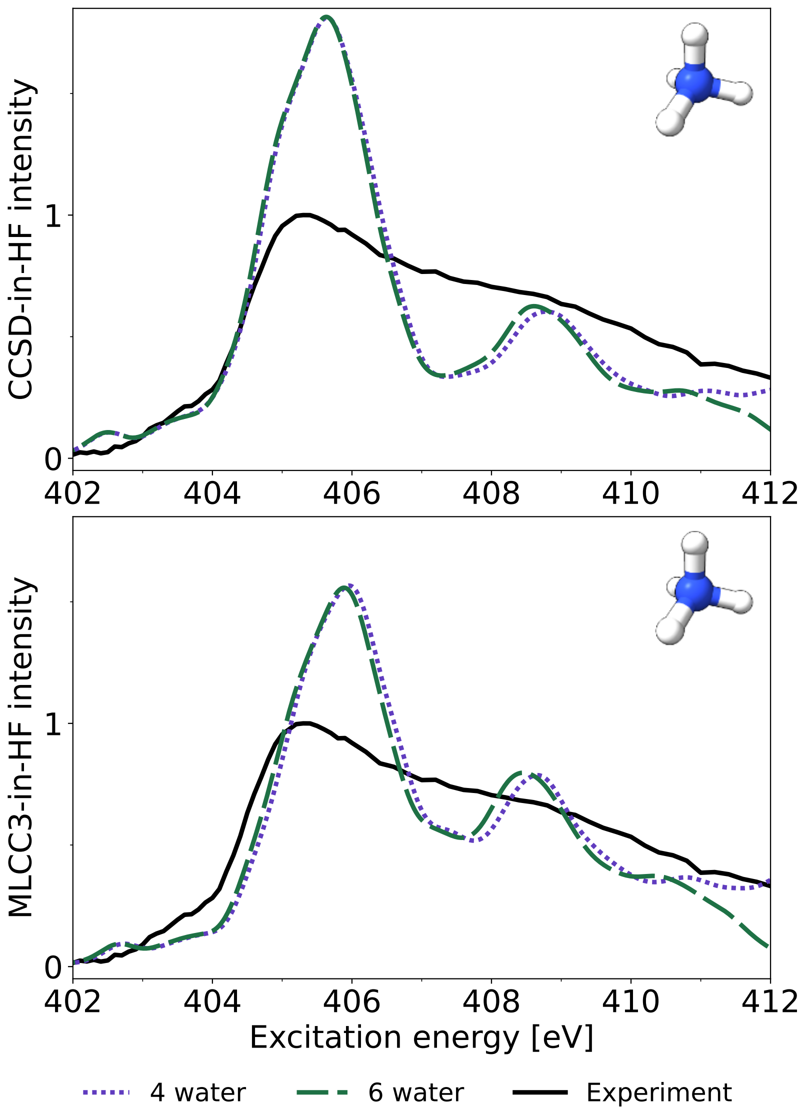

Figure S5: The effect of including 6 closest water molecules in CC-in-HF calculations for NH_4^+. Average over 30 snapshots. Voigt profiles with Lorentzian fwhm of 0.2 eV and 0.2 Gaussian standard deviation. XA spectra of **ammonium** in water clusters at the CCSD-in-HF and MLCC3-in-HF level of theory. The spectra have been shifted by $-2\,\mathrm{eV}$ and $-1\,\mathrm{eV}$, respectively.

Convergence from CCSD to CC3

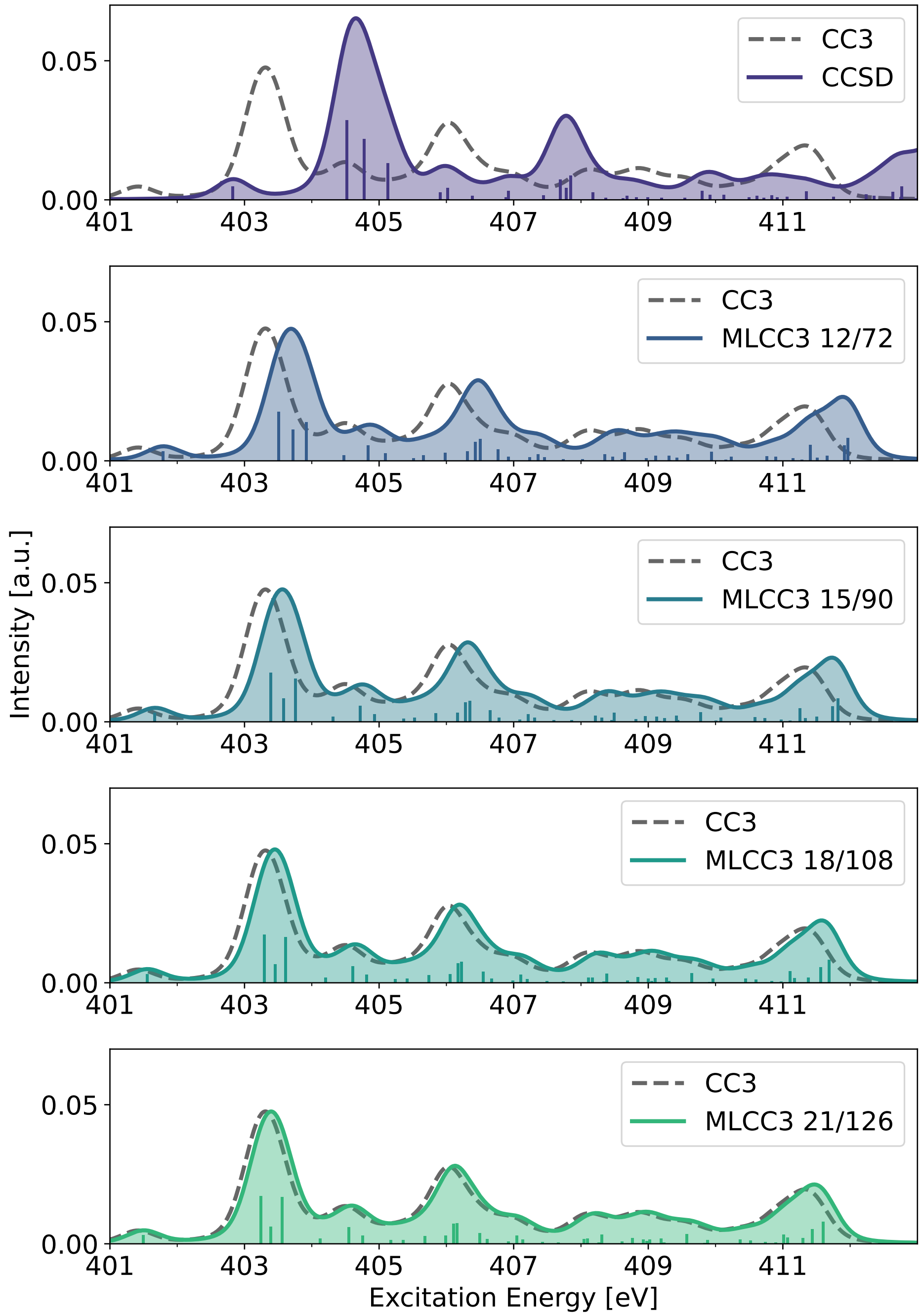

Figure S6: Convergence from CCSD to CC3 by increasing the number of occupied/virtual CNTOs included in the MLCC3 orbital space. The spectra are obtained for a single snapshot of the dynamics simulation of **ammonia** (step 2200). Individual excitations have been broadened using Voigt profiles with 0.2 eV Lorentzian fwhm and 0.2 eV Gaussian standard deviation (total fwhm ~0.59 eV).

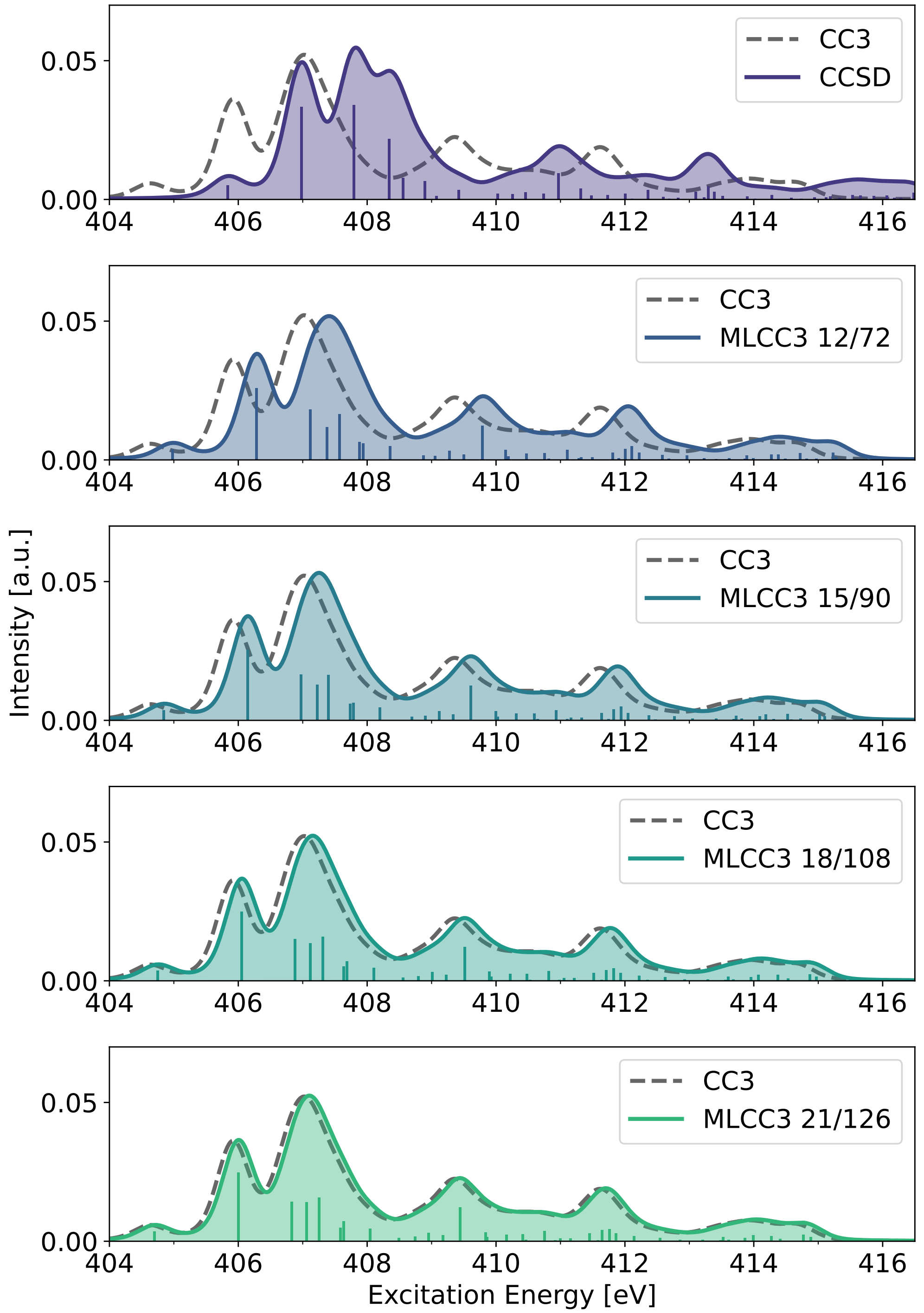

Figure S7: Convergence from CCSD to CC3 by increasing the number of occupied/virtual CNTOs included in the MLCC3 orbital space. The spectra are obtained for a single snapshot of the dynamics simulation of **ammonium** (step 2200). Individual excitations have been broadened using Voigt profiles with 0.2 eV Lorentzian fwhm and 0.2 eV Gaussian standard deviation (total fwhm ∼0.59 eV).

XA spectra using various broadening schemes

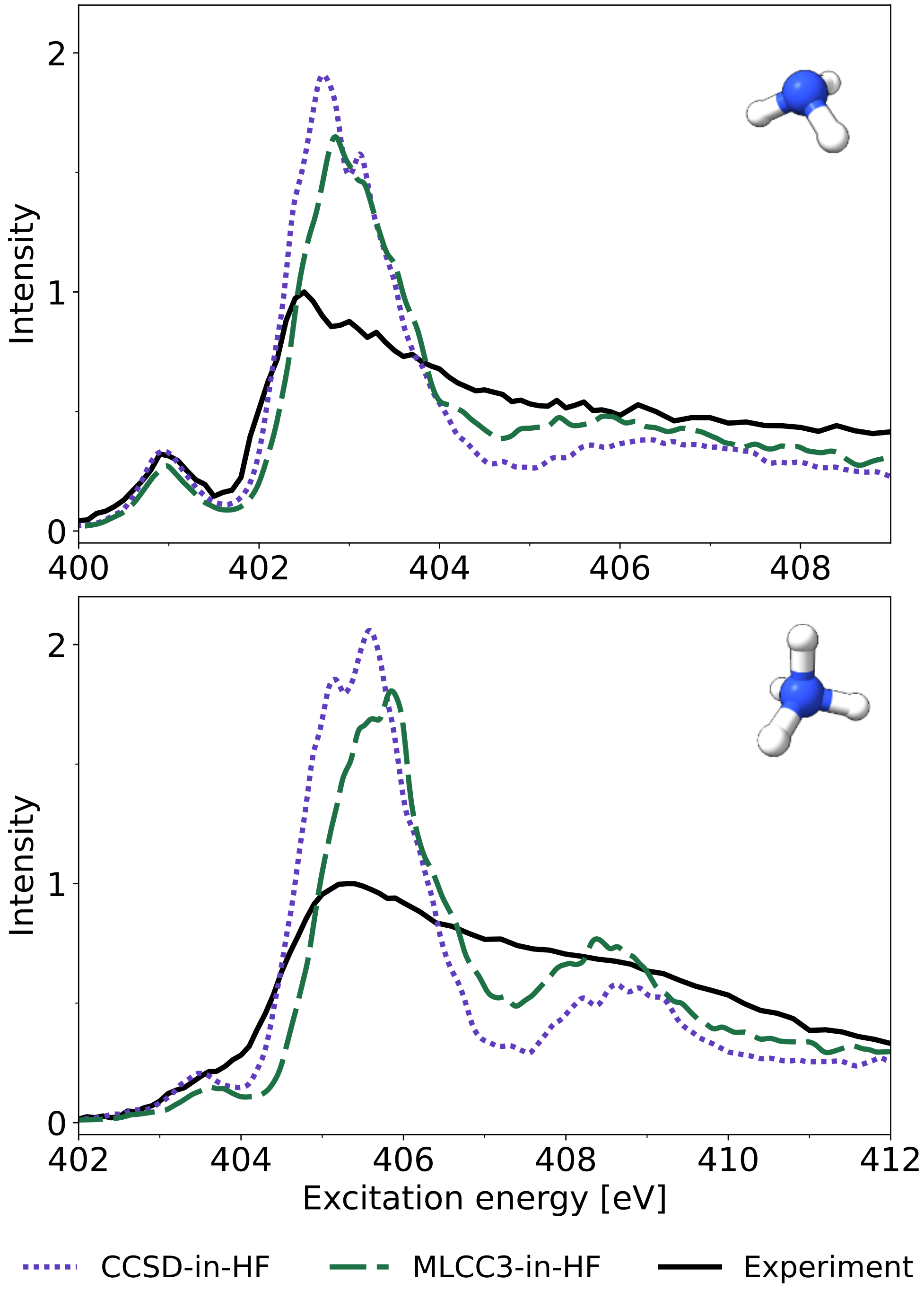

Figure S8: XA spectra of NH_3 (top) and NH_4^+ (bottom) in water clusters at the CCSD-in-HF and MLCC3-in-HF levels of theory. aug-cc-pVTZ was used for the solute, aug-cc-pVDZ for the four closest water molecules and cc-pVDZ for the remaining solvent. Broadening: Lorentzian profiles with 0.2 eV fwhm. Constant energy shifts of −2.0 eV and −1.1 eV were applied for CCSD-in-HF and MLCC3-in-HF respectively.

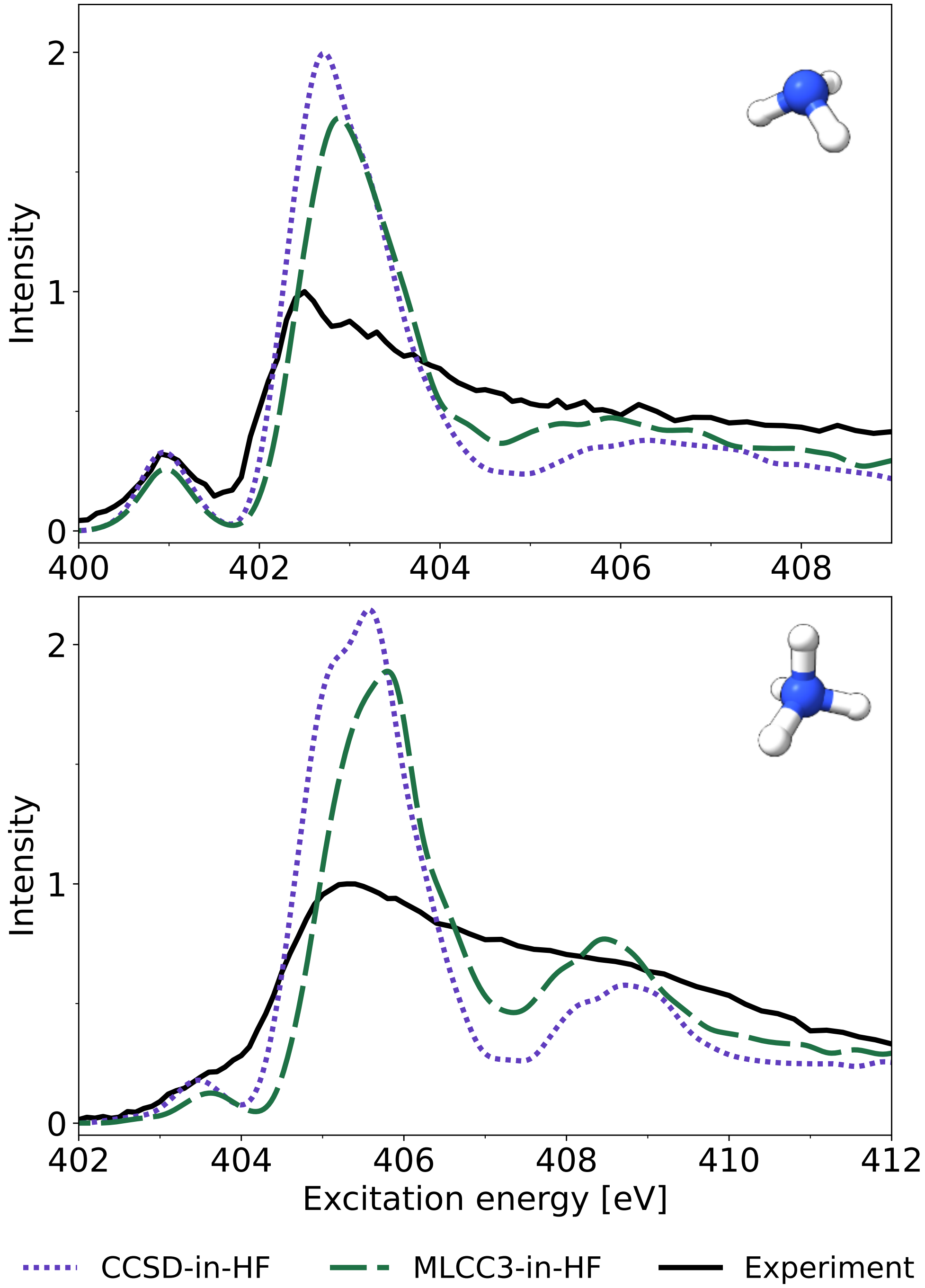

Figure S9: XA spectra of NH_3 (top) and NH_4^+ (bottom) in water clusters at the CCSD-in-HF and MLCC3-in-HF levels of theory. aug-cc-pVTZ was used for the solute, aug-cc-pVDZ for the four closest water molecules and cc-pVDZ for the remaining solvent. Broadening: Gaussian profiles with 0.4 eV fwhm. Constant energy shifts of −2.0 eV and −1.1 eV were applied for CCSD-in-HF and MLCC3-in-HF respectively.
